# Visualization of Endothelial Actin Cytoskeleton in the Mouse Retina

**DOI:** 10.1371/journal.pone.0047488

**Published:** 2012-10-24

**Authors:** Alessia Fraccaroli, Claudio A. Franco, Emanuel Rognoni, Filipa Neto, Markus Rehberg, Attila Aszodi, Roland Wedlich-Söldner, Ulrich Pohl, Holger Gerhardt, Eloi Montanez

**Affiliations:** 1 Walter Brendel Centre of Experimental Medicine and Munich Heart Alliance, Ludwig-Maximilians University Munich, Munich, Germany; 2 Vascular Biology Laboratory, London Research Institute, Cancer Research UK, Lincoln’s Inn Fields Laboratories, London, United Kingdom; 3 Max Planck Institute of Biochemistry, Martinsried, Germany; 4 Department of Surgery, Ludwig-Maximilians University Munich, Munich, Germany; West Virginia University, United States of America

## Abstract

Angiogenesis requires coordinated changes in cell shape of endothelial cells (ECs), orchestrated by the actin cytoskeleton. The mechanisms that regulate this rearrangement *in vivo* are poorly understood - largely because of the difficulty to visualize filamentous actin (F-actin) structures with sufficient resolution. Here, we use transgenic mice expressing Lifeact-EGFP to visualize F-actin in ECs. We show that in the retina, Lifeact-EGFP expression is largely restricted to ECs allowing detailed visualization of F-actin in ECs *in situ*. Lifeact-EGFP labels actin associated with cell-cell junctions, apical and basal membranes and highlights actin-based structures such as filopodia and stress fiber-like cytoplasmic bundles. We also show that in the skin and the skeletal muscle, Lifeact-EGFP is highly expressed in vascular mural cells (vMCs), enabling vMC imaging. In summary, our results indicate that the Lifeact-EGFP transgenic mouse in combination with the postnatal retinal angiogenic model constitutes an excellent system for vascular cell biology research. Our approach is ideally suited to address structural and mechanistic details of angiogenic processes, such as endothelial tip cell migration and fusion, EC polarization or lumen formation.

## Introduction

The actin cytoskeleton is composed of actin filaments (F-actin), which are constantly remodelled and are essential for the development of specialised cellular structures, such as filopodia, lamellipodia and stress fibers [1]. Reliable visualization of F-actin is therefore critical to understand the mechanisms that regulate cellular actin dynamics as well as actin-dependent cellular processes, such as morphogenesis, polarity, movement and cytokinesis [1]. The actin cytoskeleton also serves as a scaffold for signalling, a connection with the extracellular matrix (ECM) and a mechanosensor. Despite its importance, the structural organization, dynamics and regulation of F-actin in mammals *in vivo* are not well understood.

Sprouting angiogenesis, the formation of new blood vessels from pre-existing ones, is required for organogenesis and contributes to the progression of many diseases including cancer [2]. Vessel sprouting involves several morphogenetic steps, during which endothelial cells (ECs) polarize, migrate, establish cell-cell contacts and form vessel lumens [2]. These processes are critically dependent on the organization and dynamic rearrangement of the endothelial actin cytoskeleton [3], [4], [5], [6]. A lack of tools for *in vivo* imaging of F-actin structures in individual ECs in mammals has so far precluded an understanding of how ECs regulate the actin cytoskeleton during angiogenesis. Our knowledge on the organization and regulation of the endothelial actin cytoskeleton is mainly based on *in vitro* studies, which are missing essential physiological features, such as composition of the ECM, blood flow and mechanical input from the tissue [7].

Lifeact is a 17-amino-acid-long actin-binding peptide derived from yeast that specifically labels F-actin without affecting actin organization. It has been extensively used to visualize F-actin and to study actin dynamics *in vitro* and *in vivo* [8], [9]. Recently, transgenic mice ubiquitously expressing Lifeact fused to enhanced green fluorescent protein (EGFP) have been generated (Lifeact-EGFP mice), allowing visualization of F-actin in tissues and whole animals [10]. The utility of Lifeact-EGFP mice for vascular research has not been reported. Here we report an extensive analysis of the expression patterns of Lifeact-EGFP in the vascular system from the Lifeact-EGFP mice and show that: 1) in the retinas, Lifeact-EGFP is extremely useful for the analysis of endothelial actin-remodelling during vessel growth and 2) in the skin and the skeletal cremaster muscle, Lifeact-EGFP is a powerful tool for imaging vascular mural cells (vMCs). In summary, our data indicate that the Lifeact-EGFP transgenic mouse is an excellent tool for vascular cell biology research.

## Methods

### Animals

Lifeact-EGFP transgenic mice have been previously described [10]. The expression of Lifeact-EGFP is driven by a chicken actin promoter under the influence of a CMV enhancer ensuring ubiquitous expression [11]. All experiments with mice were performed in accordance to German guidelines and regulations. The protocols were approved by the Committee on the Ethics of Animal Experiments of the Ludwig-Maximilians University Munich.

### Whole Embryo Immunohistochemistry

Embryos were dissected in PBS at embryonic day (E) 10.5 and fixed overnight in fixation buffer (80% methanol, 20% DMSO). Embryos were stained as whole mounts as previously described [12].

### Analysis of Angiogenesis in the Postnatal Mouse Retina

Eyes were collected between postnatal (P) day 5 and P10 and fixed in 4% paraformaldehyde (PFA) for 2 hours at room temperature. Retinas were dissected and stained as whole mounts as previously described [13]. Table S1 details information about reagents and antibodies used for staining. Images were acquired and processed using a Leica TCS SP5 II microscope, LAS Montage Imaging software (Leica) and the IMARIS Digital Imaging software (Biplane). Wide-field images were acquired using DeltaVision OMX V3 microscope (Applied Precision) in conventional mode and Cascade II:512 EMCCD cameras (Photometrics). SoftWoRx software (Applied Precision) was used for deconvolving the images with enhanced additive method.

### Skin Immunohistochemistry

Samples were collected, fixed and stained as previously described [14].

### Immunohistochemistry and in vivo Microscopy of Skeletal Muscle

Microsurgical preparation of the cremaster muscle and *in vivo* microscopy was performed as described previously [15]. Briefly, mice were anesthetized using a ketamine/xylazine mixture (100 mg/kg ketamine and 10 mg/kg xylazine), administrated by intraperitoneal injection. The right cremaster muscle was exposed through a ventral incision of the scrotum. The muscle was opened ventrally in a relatively avascular zone, using careful electrocautery to stop any bleeding, and spread over the pedestal of a custom-made microscopy stage. Epididymis and testicle were detached from the cremaster muscle and placed into the abdominal cavity. Throughout the procedure as well as after surgical preparation during *in vivo* microscopy, the muscle was superfused with warm buffered saline. After *in vivo* microscopy, the tissue was fixed in 2% paraformaldehyde and immunostained as whole mount.

### In vivo Microscopy

The setup for *in vivo* microscopy was centered on an AxioTech-Vario 100 Microscope (Zeiss), equipped with LED excitation light (Zeiss) for fluorescence epi-illumination. Microscopic images were obtained with a water dipping objective (20x, NA 0.5) and acquired with an AxioCam Hsm camera and Axiovision 4.6 software.

## Results and Discussion

**Figure 1:**
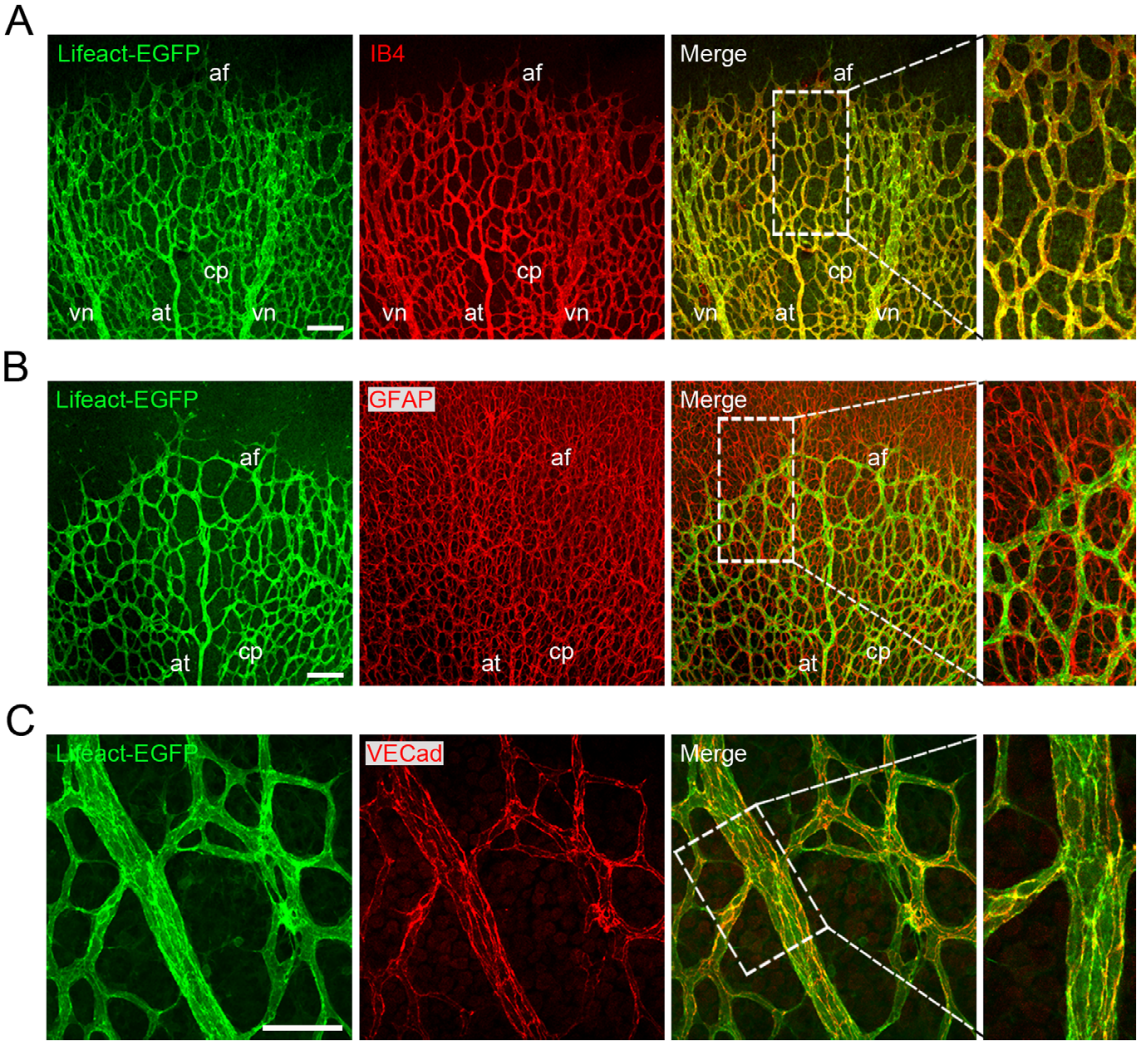
Lifeact-EGFP is highly expressed in endothelial cells but practically absent from astrocytes in the postnatal retinas of Lifeact-EGFP mice. IB4 (A in red), GFAP (B in red) or VECad (C in red) staining of whole-mounted P5 retinas. Lifeact-EGFP: green. Scale bars: A: 100 µm, B: 100 µm, C: 50 µm. af: angiogenic front; cp: central plexus; at: artery; vn: vein.

**Figure 2:**
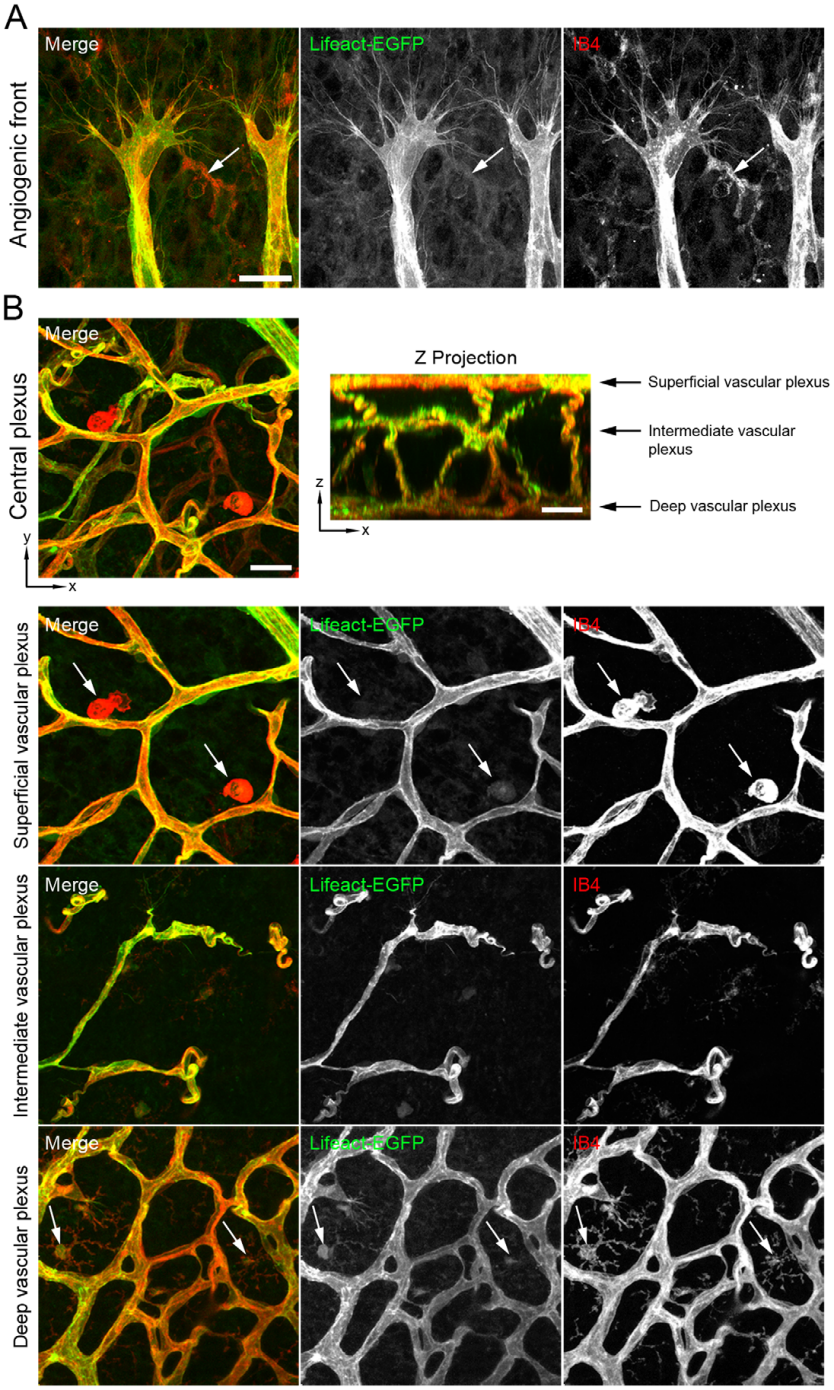
Lifeact-EGFP expression is nearly absent in retinal tissue macrophages. IB4 (in red) staining of whole-mounted P5 (**A**) and P10 (**B**) retinas. Lifeact-EGFP: green. Arrows point to IB4-positive tissue macrophages. Scale bars: 20 µm.

**Figure 3:**
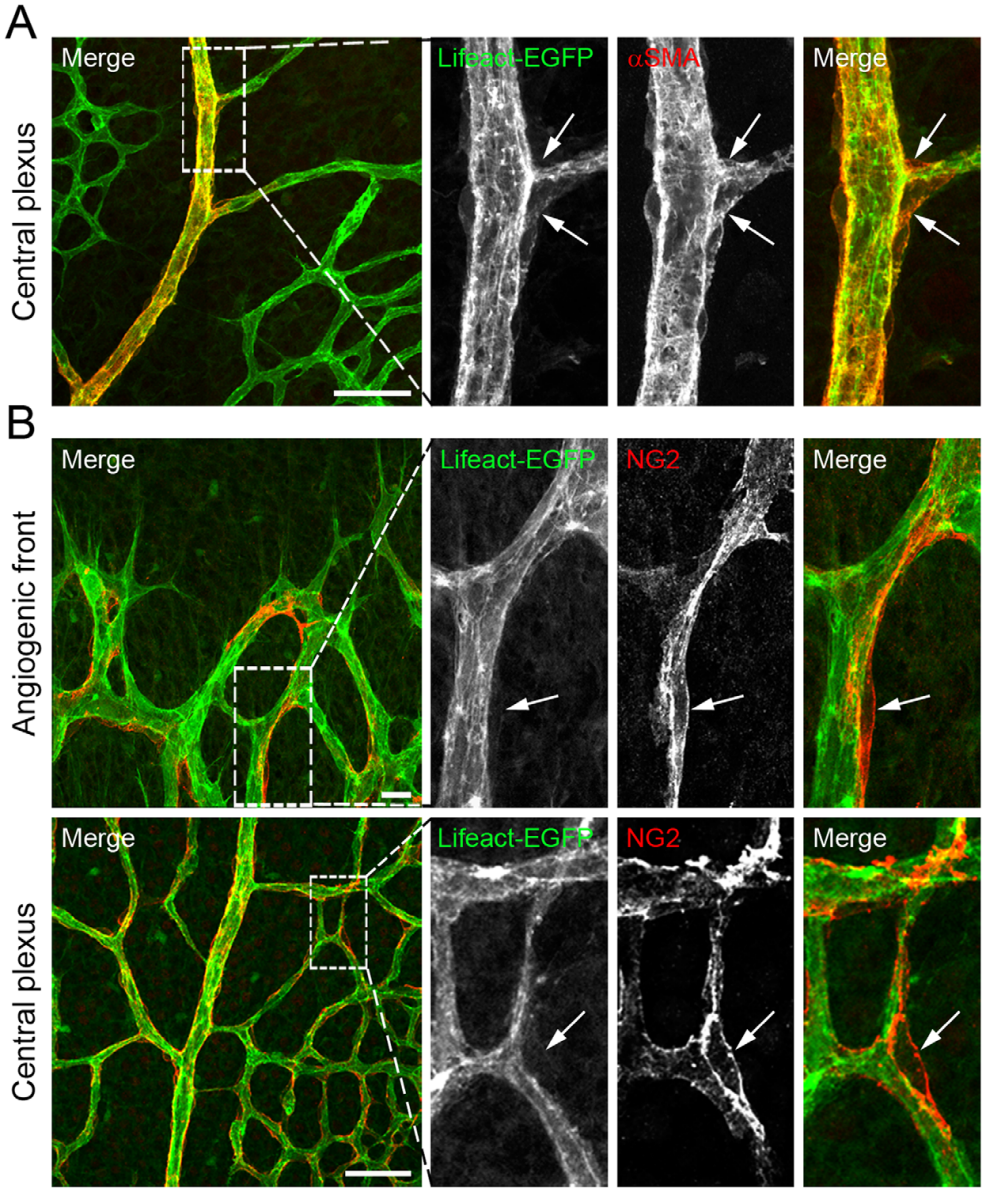
Lifeact-EGFP is weakly expressed in the vascular mural cells of the retinal vasculature. αSMA (**A** in red) or NG2 (**B** in red) staining of whole-mounted P5 retinas. Lifeact-EGFP: green. Arrows point to αSMA-positive cells (**A**) and NG2-positive pericytes (**B**). Scale bars: **A:** 50 µm, **B:** 20 µm and 50 µm.

The expression level and cellular expression pattern of Lifeact-EGFP in the vascular system of the Lifeact-EGFP mice is not known. Mouse embryos and early postnatal mouse retinas are *in vivo* models extensively used to study the mechanisms that regulate EC behaviour during angiogenesis [13]. To determine the expression of Lifeact-EGFP in ECs, we first analysed embryos and retinas of Lifeact-EGFP mice. To this end, we performed whole-mount immunostaining of E10.5 embryos with an antibody against CD31 to visualize ECs. In embryos, we find that Lifeact-EGFP is ubiquitously expressed precluding adequate imaging of F-actin structures in ECs (Figure S1). In the retina, the vascular plexus develops in close association with a pre-existing network of retinal astrocytes (ACs) [16]. To monitor Lifeact-EGFP expression in retinas, we first performed whole-mount immunostaining of retinas from postnatal day (P) 5 and P10 with isolectin B4 (IB4) to stain ECs and an antibody against glial fibrillar protein (GFAP) to visualize ACs. Lifeact-EGFP was highly expressed in ECs but practically absent from ACs (Figure 1A and B). Immunostaining of retinas with an antibody against the endothelial-specific marker vascular endothelial cadherin (VECad) further confirmed the endothelial identity of the Lifeact-EGFP-expressing cells (Figure 1C and S2). Furthermore, Lifeact-EGFP was practically absent from retinal tissue macrophages (TMs), which were positive for IB4 [17] (Figure 2). Next, we determined whether Lifeact-EGFP was expressed in retinal vascular mural cells (vMCs). For this purpose, we performed immunostaining of retinas with antibodies against alpha-smooth muscle actin (αSMA) and neuron glial antigen 2 (NG2), to visualize vascular smooth muscle cells (vSMCs) and pericytes (PCs), respectively. We found that Lifeact-EGFP was weakly expressed in vSMCs, whereas it was practically absent from PCs (Figure 3).

**Figure 4:**
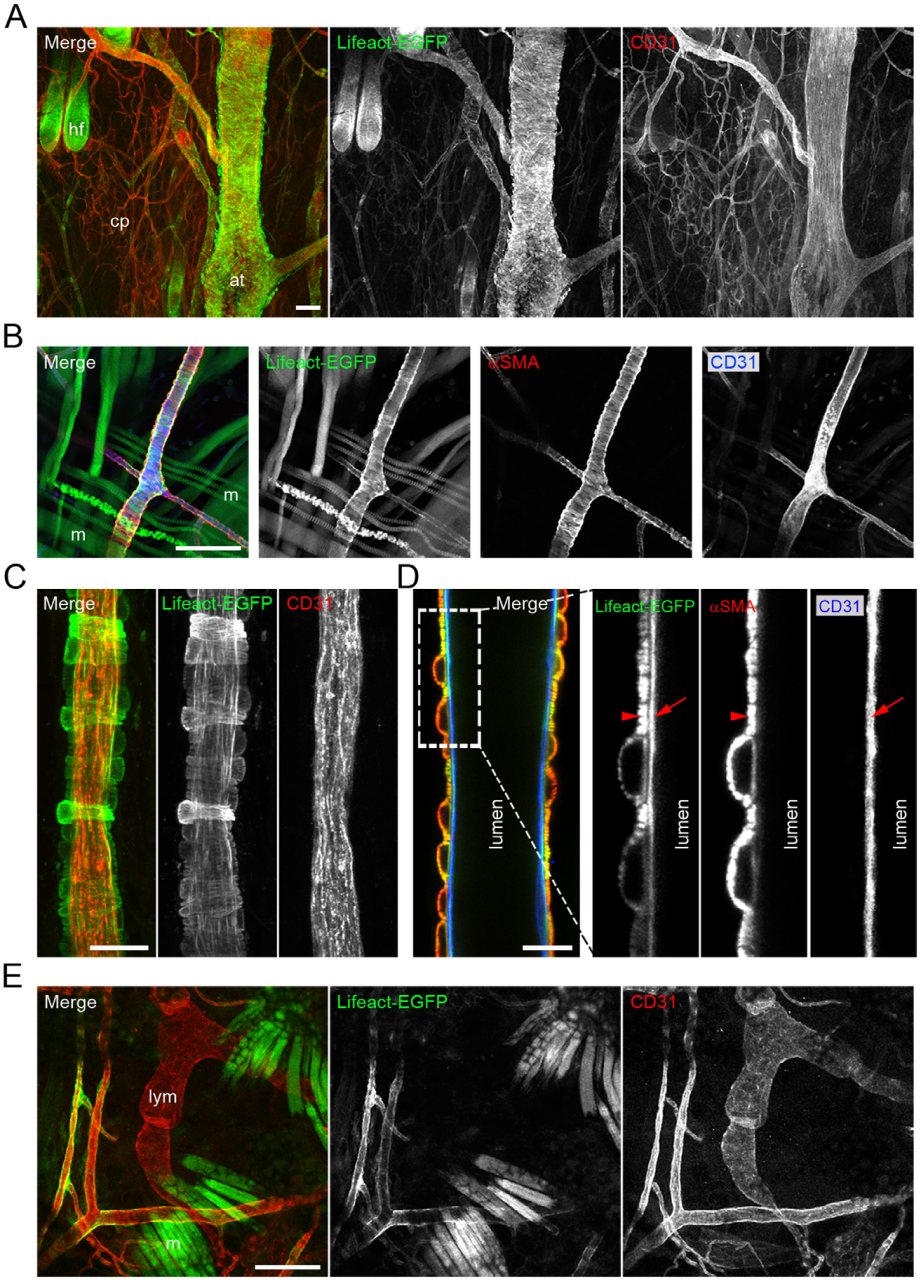
The skin and the cremaster muscle of Lifeact-EGFP mice are powerful systems to visualize F-actin of vascular mural cells. CD31 (in red) staining of whole-mounted tail skin (A) and ear (C and E). Lifeact-EGFP: green. (B and D) αSMA (in red) and CD31 (in blue) double labelling of blood vessels in the cremaster muscle. Lifeact-EGFP: green. Arrows point to endothelium and arrowheads point to vascular mural cells. Scale bars: A: 100 µm, B: 100 µm, C: 20 µm, D: 15 µm, E: 100 µm. at: artery; cp: capillaries; hf: hair follicle; lym: lymphatic endothelium; m: muscle.

Next, we addressed the expression of Lifeact-EGFP in vascular and lymphatic vessels in several organs from adult Lifeact-EGFP mice. We performed whole-mount immunostaining of skin and skeletal cremaster muscle using anti-CD31 and anti-αSMA antibodies. Contrary to the retina, we found that in the skin and the skeletal cremaster muscle, Lifeact-EGFP was highly expressed in the vMCs but weakly in ECs (Figure 4). Moreover, we were not able to detect Lifeact-EGFP in the lymphatic endothelium, suggesting that in these cells Lifeact-EGFP is almost absent (Figure 4E). The cremaster muscle is a well established *in vivo* model to study dynamic morphogenetic microvascular events such as leukocyte-EC interactions and leukocyte extravasation [18]. We observed that the leukocytes from Lifeact-EGFP mice expressed Lifeact-EGFP, enabling *in vivo* visualization of rolling and adherent leukocytes at the vessel walls of postcapillary venules (Video S1). Collectively, these results indicate that in postnatal retinas of Lifeact-EGFP mice, Lifeact-EGFP is mainly expressed in the vascular endothelium, allowing imaging of the endothelial actin cytoskeleton with excellent contrast, and that in the skin and the skeletal muscle, Lifeact-EGFP constitutes a powerful system to visualize vMCs.

**Figure 5:**
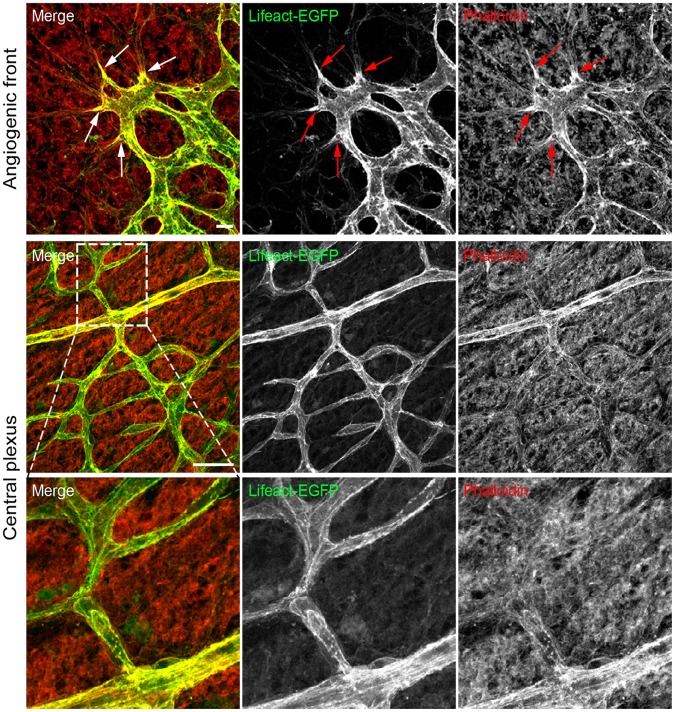
Lifeact-EGFP colocalize with phalloidin. Phalloidin (in red) staining of whole-mounted P6 retina. The leading edges of tip cells are highly enriched in actin filaments (arrows). Lifeact-EGFP: green. Scale bars: 10 and 50 µm.

During sprouting angiogenesis, ECs within the sprouts specialize into distinct functional phenotypes [2]. Tip cells initiate new sprouts, form extensive filopodia, migrate and fuse with other tip cells. The highly proliferative stalk cells follow the tip cells, elongate the stalk of the sprout, form the vessel lumen and establish cell-cell junctions to maintain sprout integrity. Finally, ECs lining the inner surface of consolidated vessels form a tight cobblestone monolayer ensuring vessel perfusion. In the postnatal retina, all stages of the sprouting process can be efficiently analysed in a single sample owing to the spatiotemporal sequence of angiogenic network formation [13]. To characterize Lifeact-EGFP expression and to visualize F-actin in vascular ECs *in situ*, we first stained whole-mounted retinas with fluorescent phalloidin, an F-actin binding compound that labels retinal vascular and non-vascular structures [19]. In agreement with previously published data [19], phalloidin mainly stained the angiogenic front of the vascular plexus where it highlighted leading edges and filopodia of the tip cells (Figure 5). The weak phalloidin stain in the ECs at the central part of the vascular plexus, as well as the label of non-vascular structures make the visualization of the endothelial actin cytoskeleton in consolidated vessels difficult (Figure 5). Importantly, Lifeact-EGFP overlapped with phalloidin at the leading edges of the vascular plexus and at the filopodia of tip cells (Figure 5). However, in contrast to phalloidin stain, Lifeact-EGFP signal was bright in the entire vascular plexus, enabling visualization of F-actin in all types of specialized ECs (Figure 5). Together, our results show that in the retina Lifeact-EGFP labels endothelial F-actin in higher grade and better signal-to-noise ratio compared to phalloidin.

**Figure 6:**
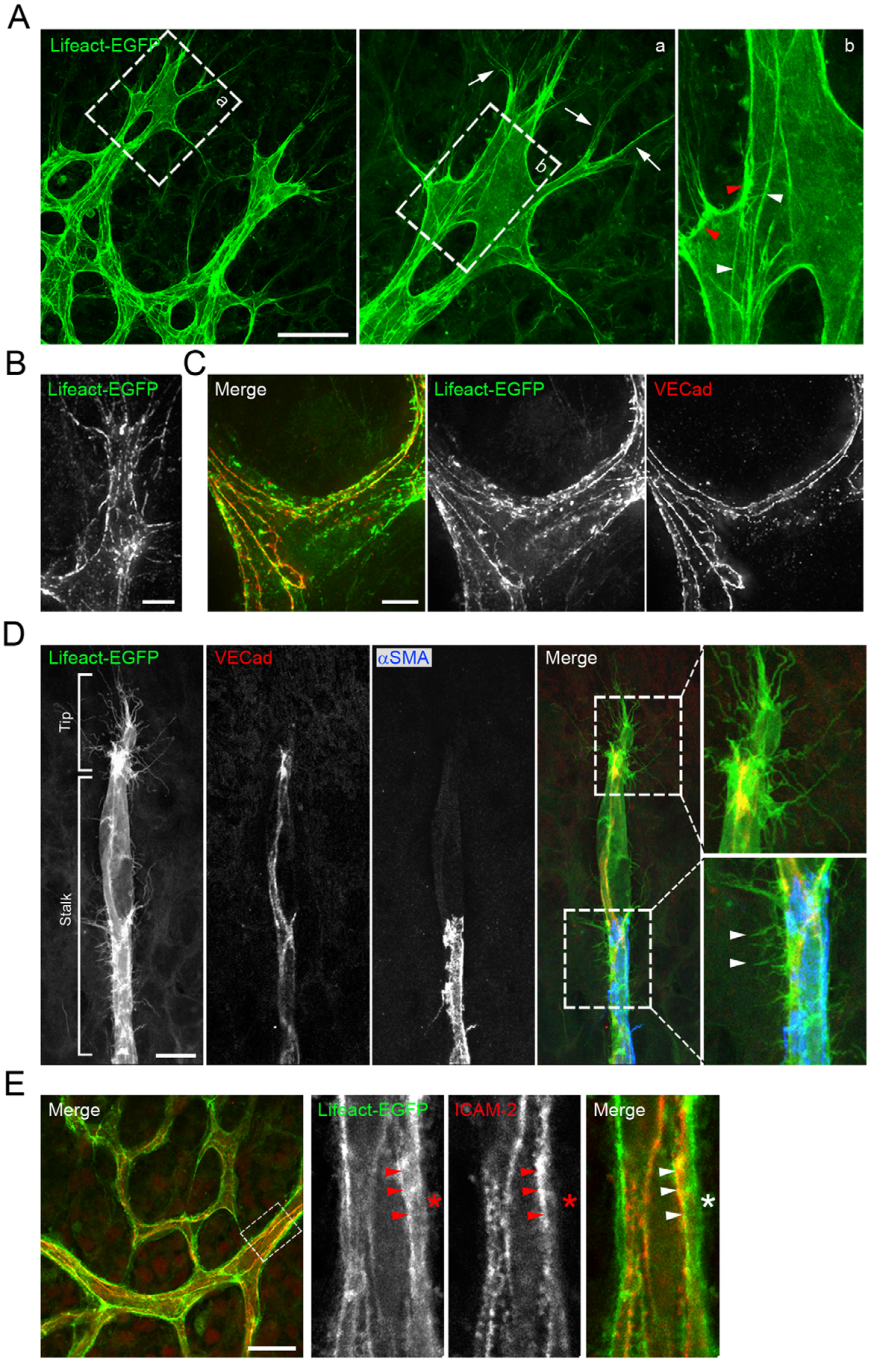
Lifeact-EGFP labels cortical actin, filopodia and cytoplasmic actin bundles in tip and stalk endothelial cells. (**A**) Migrating tip cells. Arrows indicate tip cell filopodia, white arrowheads point to F-actin filaments in the cytoplasm and red arrowheads point to cortical actin. High-magnification deconvolution images of tip cell (**B**) and stalk cells (**C**) showing details of actin cytoskeleton organization (Lifeact-EGFP: green), with fine actin-rich filopodia protrusions and cortical actin cables, running along adherens junctions (VECad: red) (**D**) VECad (in red) and αSMA (in blue) double labelling of a vascular sprout. Lifeact-EGFP: green. Arrowheads indicate short actin filament protrusions in stalk cells. (**E**) Optical section of retinal vasculature stained with ICAM-2 (in red). Lifeact-EGFP: green. Arrowheads point to the luminal endothelial membrane and the asterisk marks the abluminal side of a vessel. Scale bars: **A**: 30 µm, B: 5 µm, C: 5 µm, D: 10 µm, E: 20 µm.

The planar outgrowth of the inner vasculature in the retina allows high-resolution three-dimensional imaging of the endothelium [7]. To visualize and characterize the actin cytoskeleton in ECs, we performed high-resolution confocal laser scanning microscopy (Figure 6A, D and E) and high-power deconvolution imaging (Figure 6B and C) of sprouting vessels in P6 retinas from Lifeact-EGFP mice. We found that in migrating tip cells, Lifeact-EGFP brightly labelled cortical actin, filopodia as well as long and thin actin bundles in the cytoplasm resembling stress fibers (Figure 6A and B). In stalk cells, Lifeact-EGFP highlighted endothelial junctions (Figure 6C) and short filopodial-like protrusions along the cell membrane (Figure 6D). Lifeact-EGFP demarcation allowed a morphometric analysis of these actin protrusions; they had a length of 3.5 µm ±0.61 µm (n = 50). Similar protrusions were also observed in EC in the established plexus (Figure S2) and in anastomosing tip cells at the fusion points (Figure S3). Next, we labelled lumenized vessels with an antibody against intercellular adhesion molecule 2 (ICAM-2), a marker for the luminal EC membrane [20]. We found that Lifeact-EGFP partially localizes adjacent to ICAM-2 stain (Figure 6E), indicating that Lifeact-EGFP marks F-actin along the luminal as well as the abluminal membrane of the endothelium. This localization pattern is well suited for future studies of EC apical-basal polarity. Together, our results using Lifeact-EGFP identified actin-based protrusions and filopodia, actin-enriched cell membrane regions and cytoplasmic F-actin structures in ECs within sprouting and consolidated vessels.

In summary, the results presented here show that the Lifeact-EGFP mouse in combination with the early postnatal retinal angiogenic model is an excellent system to visualize and characterize the actin cytoskeleton in individual ECs *in situ*. We believe that these mice will be a great resource for vascular research and can be used for the study of morphogenetic angiogenic processes such as migration, polarization and anastomosing of ECs as well as lumen formation.

## Supporting Information

Figure S1 **Lifeact-EGFP is ubiquitously expressed during embryogenesis.** CD31 (in red) staining of whole-mounted E10.5 transgenic Lifeact-EGFP embryos. Lifeact-EGFP: green. (**A**) 3-dimensional reconstruction images. (**B**) Confocal sections. Arrows point to intersomitic vessels and arrowheads indicate epithelial actin-rich apical site. Scale bars: 50 µm. Ep: Epithelial cell; Me: Mesenchymal cell; NT: Neural tube.
(TIF)

Figure S2 **Lifeact-EGFP expression is practically absent in retinal astrocytes.** GFAP (in red) and VECad (blue) double labelling of retinas. Lifeact-EGFP: green. Arrowheads indicate short actin filament protrusions in ECs. Scale bars: 20 µm.
(TIF)

Figure S3 **Confocal images of endothelial actin cytoskeleton in retinal vasculature.** **(A and B)** Visualization of endothelial actin cytoskeleton (Lifeact-EGFP: white) during anastomosing of tip cells. Arrowheads point to short actin filament protrusions during the tip cell fusion process. **(C)** 3-dimensional reconstruction of an EC sprout. Lifeact-EGFP: white. Arrow points to an actin filament protrusion. Scale bars: 20 µm.
(TIF)

Table S1 **Antibodies and reagents used for staining.**
(DOCX)

Video S1 **Postcapillary venules in the cremaster muscle from Lifeact-EGFP mouse.**
(AVI)
