# Supplementary figures and images for: Visualization of Endothelial Actin Cytoskeleton in the Mouse Retina
Source: PLoS One. 2012 Oct 24;7(10):e47488. doi: 10.1371/journal.pone.0047488 (PMC3480364; doi:10.1371/journal.pone.0047488)

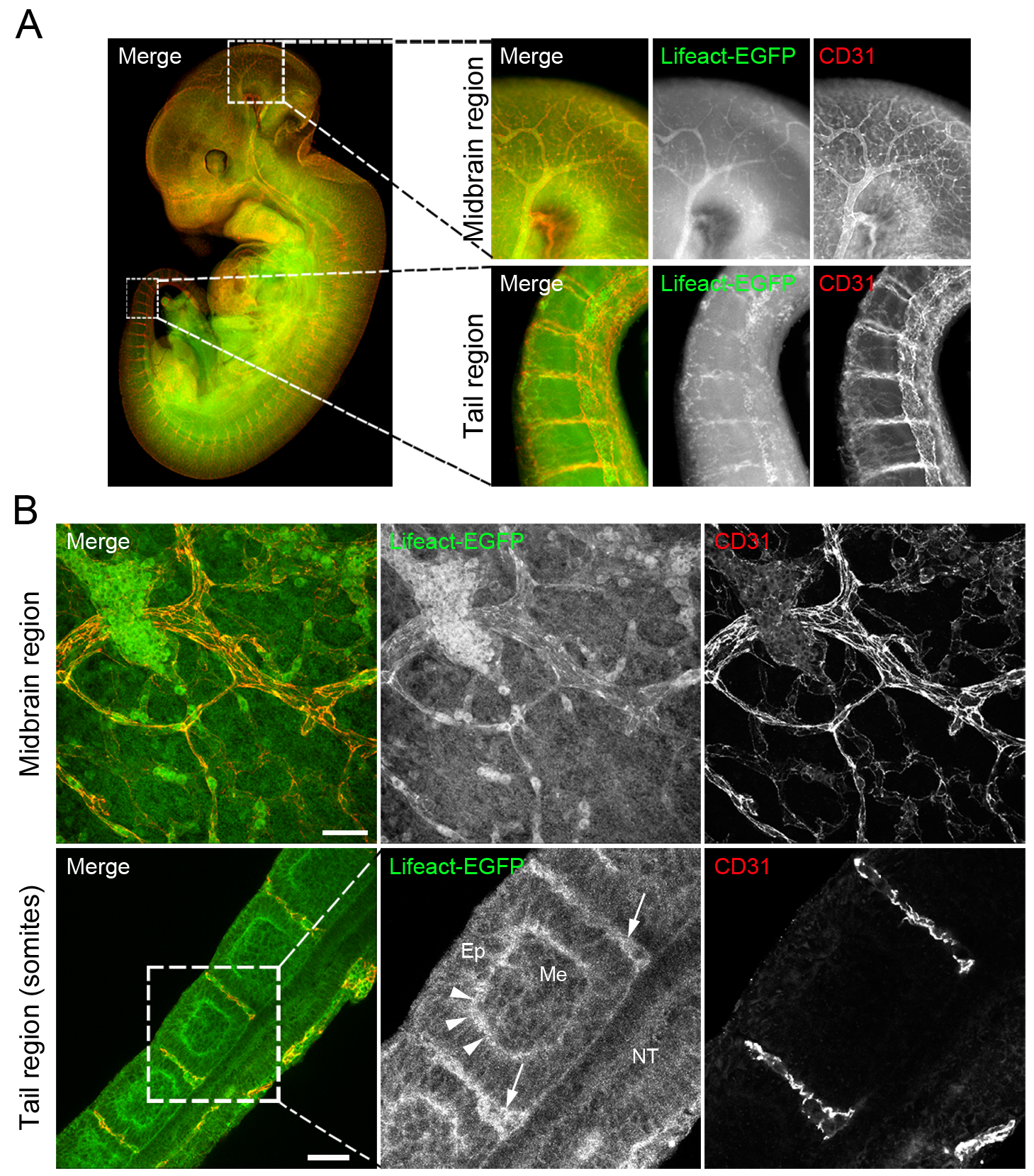

Supplement: Figure S1 — Lifeact-EGFP is ubiquitously expressed during embryogenesis. CD31 (in red) staining of whole-mounted E10.5 transgenic Lifeact-EGFP embryos. Lifeact-EGFP: green. (A) 3-dimensional reconstruction images. (B) Confocal sections. Arrows point to intersomitic vessels and arrowheads indicate epithelial actin-rich apical site. Scale bars: 50 µm. Ep: Epithelial cell; Me: Mesenchymal cell; NT: Neural tube. (TIF) [file pone.0047488.s001.tif]

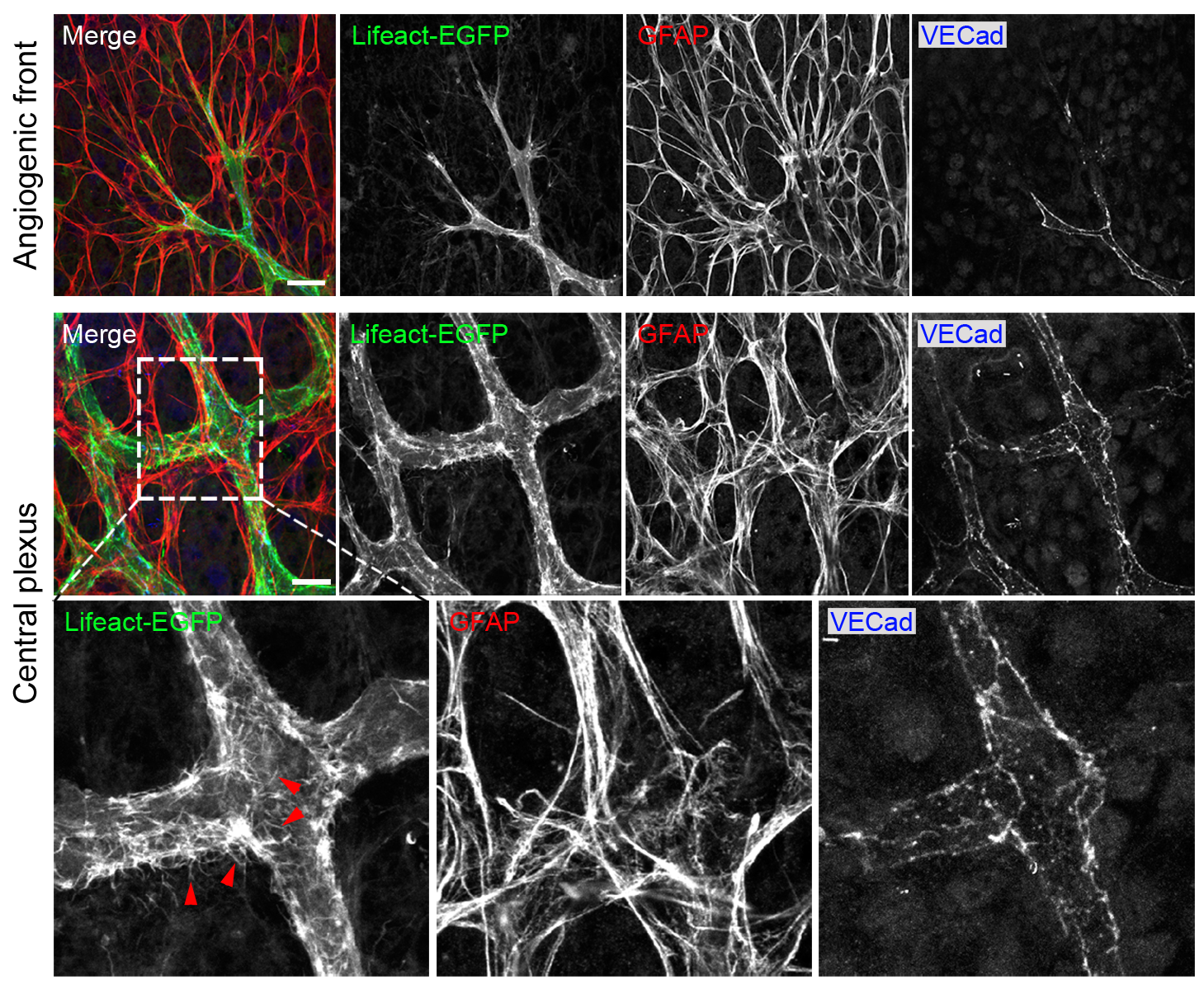

Supplement: Figure S2 — Lifeact-EGFP expression is practically absent in retinal astrocytes. GFAP (in red) and VECad (blue) double labelling of retinas. Lifeact-EGFP: green. Arrowheads indicate short actin filament protrusions in ECs. Scale bars: 20 µm. (TIF) [file pone.0047488.s002.tif]

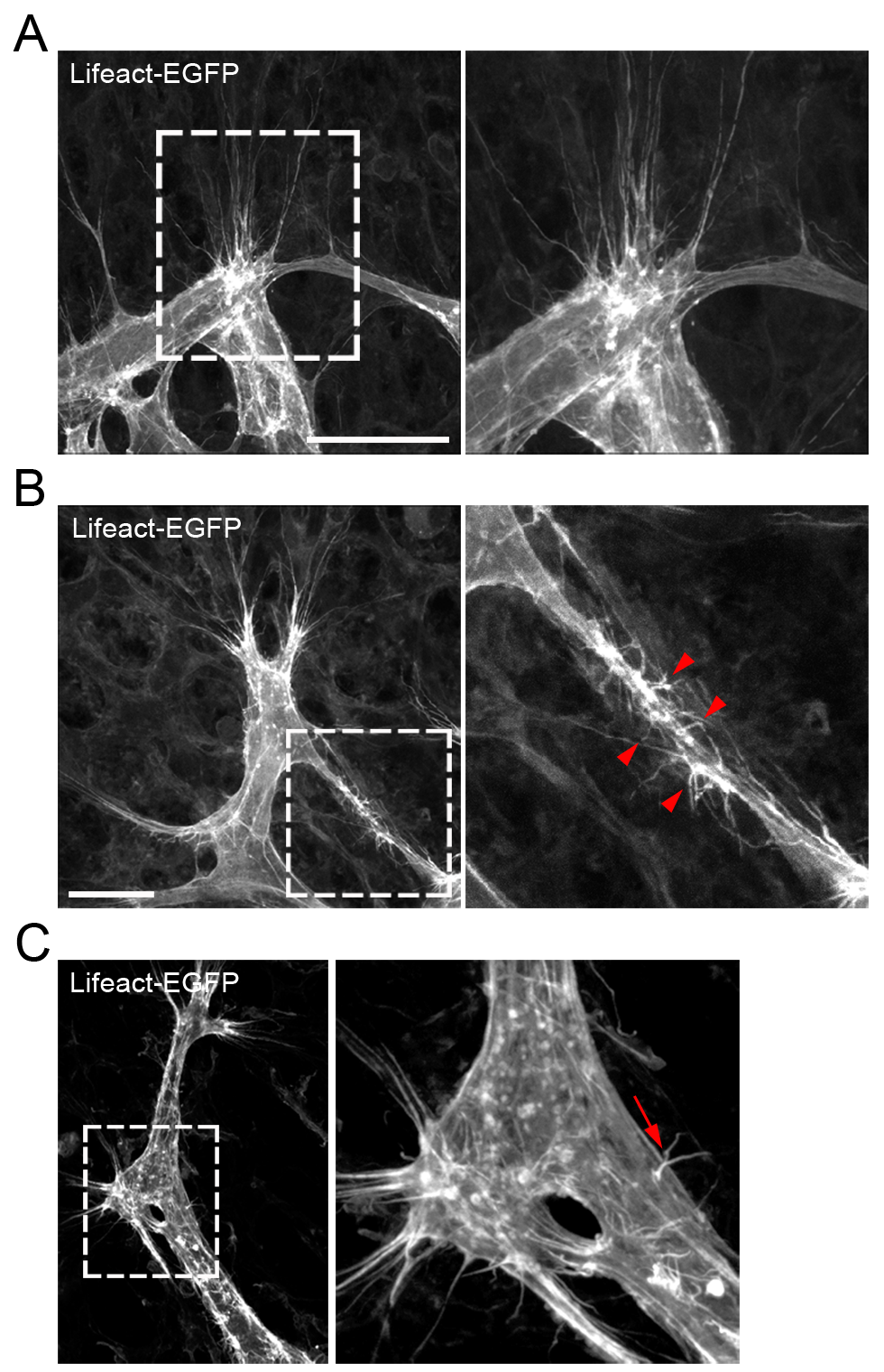

Supplement: Figure S3 — Confocal images of endothelial actin cytoskeleton in retinal vasculature. (A and B) Visualization of endothelial actin cytoskeleton (Lifeact-EGFP: white) during anastomosing of tip cells. Arrowheads point to short actin filament protrusions during the tip cell fusion process. (C) 3-dimensional reconstruction of an EC sprout. Lifeact-EGFP: white. Arrow points to an actin filament protrusion. Scale bars: 20 µm. (TIF) [file pone.0047488.s003.tif]
