# Supplementary material for: Visualization of Endothelial Actin Cytoskeleton in the Mouse Retina
Source: PLoS One. 2012 Oct 24;7(10):e47488. doi: 10.1371/journal.pone.0047488 (PMC3480364; doi:10.1371/journal.pone.0047488)
Supplement: Table S1 — Antibodies and reagents used for staining. (DOCX) [file pone.0047488.s004.docx]

**Table S1: Antibodies and reagents used for staining.**

| **Antibody/Reagents** | **Concentration** | **Vendor (Catalog number)** |
| --- | --- | --- |
| Anti-GFAP | 1:75 | Dako, (Z0334) |
| Anti-VECad | 1:100 | eBioscience, (14-1442) |
| Anti-NG2 | 1:100 | Chemicon, (AB5320) |
| Anti-CD31 | 1:100 | BD Pharmingen, (553370) |
| Anti-αSMA | 1:100 | Sigma, (C6198) |
| Anti-ICAM-2 | 1:200 | BD Pharmingen, (553326) |
| Biotinylated *Griffonia simplicifolia* lectin I (IB4) | 1:50 | Vector Laboratories, (B-1205) |
| Phalloidin-TRITC | 1:150 | Sigma, (P1951) |
